# Evaluation of Protection Level, Respiratory Safety, and Practical Aspects of Commercially Available Snorkel Masks as Personal Protection Devices Against Aerosolized Contaminants and SARS-CoV2

**DOI:** 10.3390/ijerph17124347

**Published:** 2020-06-19

**Authors:** Peter Germonpre, Dirk Van Rompaey, Costantino Balestra

**Affiliations:** 1 Centre for Hyperbaric Oxygen Therapy, Military Hospital Brussels, 1120 Brussels, Belgium; 2 DAN Europe Research Department, 64026 Roseto, Italy; costantinobalestra@gmail.com; 3 Defence Laboratories DLD, Kwartier Maj Housiau, 1800 Vilvoorde, Belgium; dirk.vanrompaey@mil.be; 4 Environmental & Occupational, Ageing (Integrative) Physiology Lab, Haute Ecole Bruxelles-Brabant (HE2B), 1160 Auderghem, Belgium; 5 Motor Sciences Department, Physical Activity Teaching Unit, Université Libre de Bruxelles (ULB), 1050 Brussels, Belgium

**Keywords:** SARS-CoV2, personal protection devices, snorkel masks, safety test

## Abstract

*Introduction*: The “Severe Acute Respiratory Syndrome Coronavirus 2″ (SARS-CoV2) pandemic has led to a worldwide shortage of personal protection devices (PPD) for medical and paramedical personnel. Adaptation of commercially available snorkel masks to serve as full face masks has been proposed. Even not formally approved as PPD, they are publicized on social media as suitable for this use. Concerns about actual protection levels and risk of carbon dioxide (CO_2_) accumulation while wearing them for extended periods made us perform a systematic testing of various brands, in order to verify whether they are as safe and effective as claimed. *Methods*: A ‘fit’ test was performed, analogous to gas mask testing. Respiratory safety was evaluated by measuring end-tidal CO_2_ and oxygen saturation while wearing the masks in rest and during physical exercise. Masks were tested with 3D adaptors to mount regular bacterial-viral ventilator filters when available, or with snorkel openings covered with N95/FFP2 cloth. *Results*: Modified masks performed reasonably well on the fit test, comparable to regular N95/FFP2 masks. Not all ventilator filters are equally protective. For all masks, a small initial increase in end-tidal CO_2_ was noted, remaining within physiological limits. 3D printed adaptors are safer, have more flexibility and reliability than makeshift adaptations. *Conclusions*: These masks can offer benefit as a substitute for complete protective gear as they are easier to don and remove and offer full-face protection. They may be more comfortable to wear for extended periods. Proper selection of mask size, fit testing, quality of 3D printed parts, and choice of filter are important.

## 1. Introduction

Early 2020, infection with SARS-CoV2 (Severe Acute Respiratory Syndrome Coronavirus 2) rapidly spread from East Asia to the rest of the world and from end of February took pandemic proportions [1]. In many countries, hospitals were overwhelmed with patients presenting severe respiratory distress syndrome and as the mode of transmission consists primarily of droplet and aerosol viral particle spread, personal protection devices (PPD) such as respiratory masks, face shields, and protective clothing are crucial in protecting healthcare workers [2]. As these PPD are almost exclusively of the disposable type, the manufacturing and supply lines were soon exhausted or faced cross-border transportation difficulties [3]. Moreover, the correct donning and doffing of full protective gear is a time-consuming process, and working in full protective dress for prolonged or repeated periods is exhausting and may lead, in the case of respiratory masks, to pressure-induced lesions in the face [4].

Creative thinking resulted in the adaptation of commercially available snorkel masks for use as full-face protection devices in this setting. Various 3D printed adaptors have been announced in rapid succession, converting snorkel masks into positive-pressure, non-invasive ventilatory support masks for patients (which will not be considered here), or PPD for use by medical personnel.

The theoretical advantages of using such masks instead of an assembly of oro-nasal protection mask and protective goggles, are ease of use, reusability, and possibly better wearer comfort. Possible disadvantages could be insufficient protection level, increased work of breathing, carbon dioxide (CO_2_) accumulation, reduced communication, fogging of the face shield.

Even though no formal evaluation has been done of these masks and various types of adaptations, makeshift or more professional, the use of these masks in hospital emergency wards and intensive care wards has already spread widely, at least in certain countries or regions [5,6,7].

Concerns about the actual protection level offered by these masks and the risk of carbon dioxide (CO_2_) accumulation while wearing them for extended periods made us perform a systematic testing of various brands, in order to verify whether they are as safe and effective as claimed.

For use as a personal respiratory protection device against air- or aerosol-borne bacteria and viruses in a ‘high-load’ environment such as healthcare workers in direct contact with infected patients, face masks or respirators should comply with the N95/FFP2 or even N99/FFP3 standard [8]. These standards ensure that 95% (N95/FFP2) or 99% (N99/FFP3) of non-oil-based particles such as those resulting from wildfires, air pollution, volcanic eruptions, or bioaerosols (e.g., viruses) are filtered out [9].

## 2. Materials and Methods

Snorkel masks and, if available, 3D printed accessories, were acquired from local dive stores, or obtained directly from the manufacturer (Subea and Ocean Reef).

Each mask was assembled for use as personal protection device using the 3D printed accessory, if and when such instructions and accessories were available. After fitting the adaptor to the snorkel attachment, a 22 mm connector, standard hospital respiratory filter, can be connected. The choice of filter will obviously depend on the local availability. Most filters in use as ventilatory protection or combined with airway humidification have a bacterial and viral filtration efficiency of 99.999%. While the precise configuration and mode of filtration of various brands of filters may vary (electrostatic versus pleated mechanical filters), all filters are for ventilatory use considered equally effective, differing mainly in the resistance to air flow. A review and extensive testing of a large number of commercially available filters was published in 2004 [10]; specifications of each filter brand and type are available from the manufacturer. The 3D part for the Ocean Reef mask also allows for the connection of a standard 40 mm threaded filter, for which a P3 grade filter was supplied by the manufacturer. Other filters were chosen from the commonly used filters in our hospital.

If no 3D printed parts were available, the snorkel was covered with a single layer of protective cloth obtained from a regular disposable protective face mask (FFP2 or N95 grade) [9], carefully taped with plastic cello-tape. This procedure was based on information obtained from healthcare workers in various hospitals in Belgium and The Netherlands as a workable solution (personal communication). On the Subea mask it was possible to remove the snorkel ball/float, which impedes water entry while snorkeling, to increase the air entry opening.

**Figure 1:**
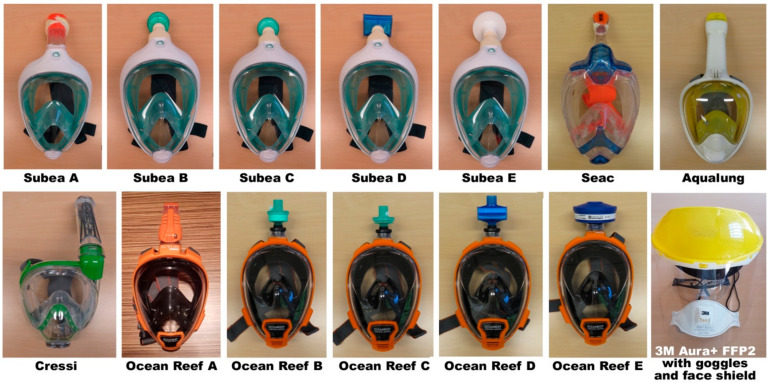
Snorkel mask configurations tested. See Table 1 for details. Lower right: ‘full face’ assembly with FFP2 mask (3M Aura 9322+), protection goggles and face shield, for size comparison.

**Figure 2:**
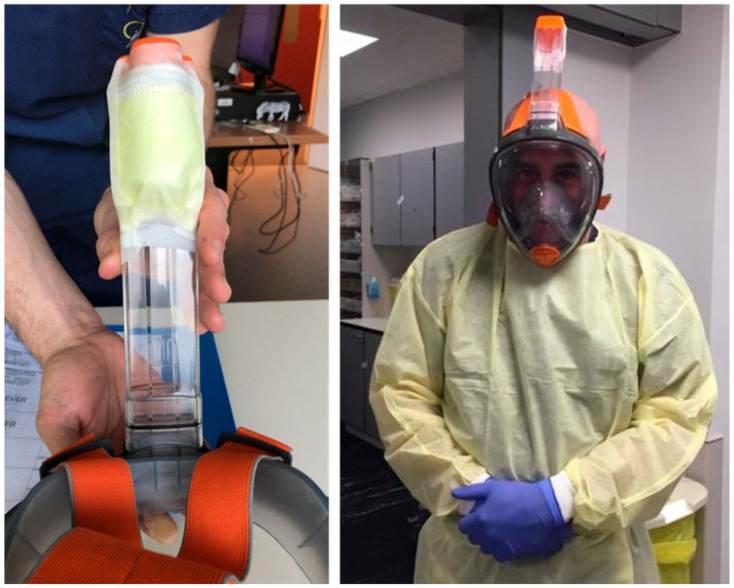
Snorkel mask ‘makeshift’ configuration with FFP2 cloth (photos courtesy M. Gillis).

A number of configurations were thus obtained, and evaluated according to protection level, respiratory safety, and practical use (Figure 1 and Figure 2, Table 1).

### 2.1. Protection Level

Protection level was measured by ‘fit testing’, analogous to the fitting of gas masks and dedicated protective masks. Fit testing was performed using a particle counter (TSI PortaCount Pro+ 8038; TSI Incorporated, Shoreview, MN, USA), simultaneously measuring particle numbers in- and outside the mask. This results in a ‘fit factor’, the proportion of particles blocked.

Fit Factor = C out/C in(1)

Particle counting was performed at the Belgian Defence Laboratories (DLD). In order to minimize variability, a single test person was used for all masks. Mask size was chosen to fit the test person’s face optimally according to the manufacturers’ instructions. In most cases, these masks are available in only two sizes, S/M and M/L, using the chin to eye-pupil distance as a measure of mask size. According to the particle counter instructions and tools, a sampling nipple was mounted in an unobtrusive location on the mask’s silicone side skirt, a second nipple on the inside skirt, and a small sampling tube was fitted so the sampling took place immediately in front of the nose and mouth.

**Figure 3:**
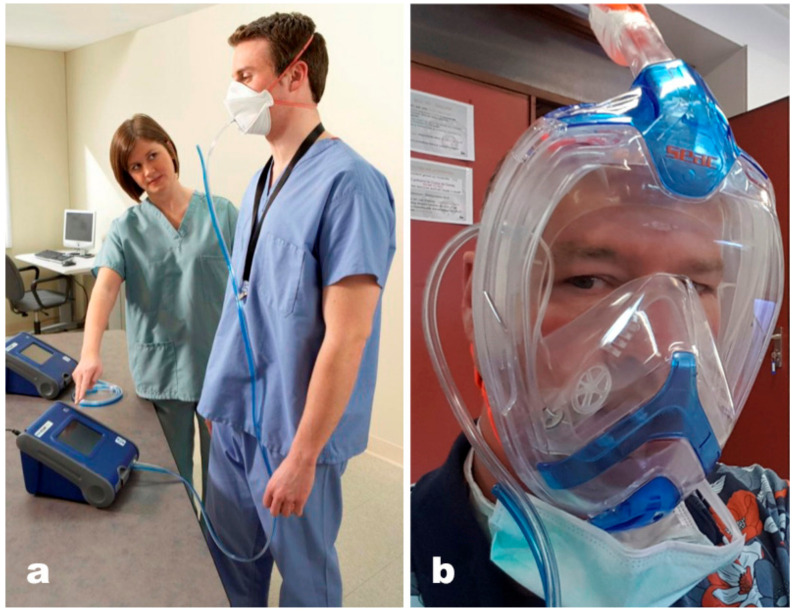
Particle count sampling setup. (**a**) (from [12]) general setup; (**b**) sampling tube fitted inside snorkel mask towards nose and mouth breathing chamber. Blue tubing—ambient air sampling line, transparent tubing—mask sampling line.

Air was sampled by the PortaCount Pro+ from inside the mask as well as from immediately outside the mask (Figure 3) and analyzed in a miniature continuous-flow Condensation Nucleus Counter (CNC). A CNC takes particles that are too small to be easily detected, grows them to a larger, easily detectable size, and then counts them [11]. The PortaCount Pro+ CNC grows submicron particles to supermicron alcohol droplets and then measures the concentration of the alcohol droplets. This makes the PortaCount Pro+ Fit Tester sensitive to particles with diameters as small as 0.015 microns, but insensitive to variations in particle size, shape, composition, and refractive index. Thus, quantitative fit testing can be performed with virtually any aerosol, including ambient air. The range of particles that can be detected spreads from 0.015 to 1 micrometer [12]. The testing protocol (described below) is approved by the Occupational Safety and Health Administration (OSHA) [13].

**Figure 4:**
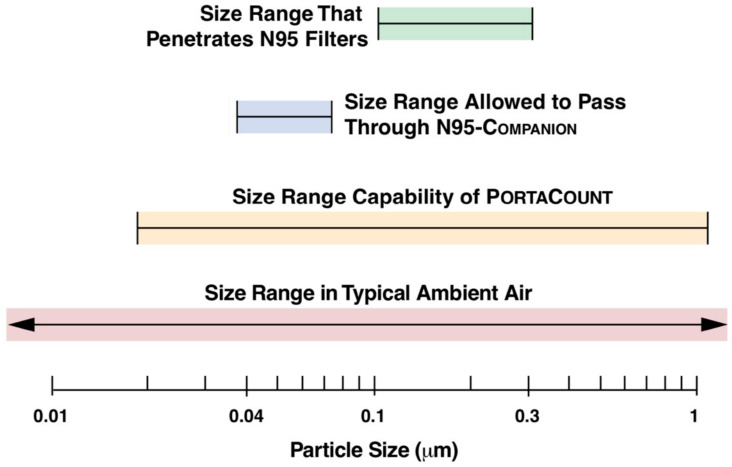
Principle of N95 Companion Protocol (modified from [15]).

Fit testing using particles requires the filter to be maximally effective, so that the Fit Factor reflects the seal between mask and face. For filters that do not comply with the 99% filter efficiency (N99 or FFP3 standard), obviously, many particles would be counted inside the mask that have passed through the filter. In order to eliminate this bias, the PortaCount Pro+ 8038 can be instructed to use a ‘N95 Companion Protocol’, whereby only a subset of particle sizes (negatively charged particles of 40–70 nanometers) are counted. Before particle counting, sampled air is passed through a filter which blocks all particles except those in the 40–70 nanometer size range. As this is the particle size which is most efficiently filtered out by N95/FFP2 class filters, any particles detected using this protocol are considered to have passed through the face seal, not the filter (see Figure 4). The choice of acceptable Fit Factor may vary, however, for half-masks a minimal Fit Factor of 100 is considered (meaning that only 1% of particles in outside air is present inside the breathing mask) [14]. Using the N95 protocol, the maximum Fit Factor calculable is 200.

First, a global mask adjustment test (‘Total Inward Leakage’ or TIL) was performed, both with the ‘standard protocol’ and then with the ‘N95 protocol’, to ascertain the absence of major leaks and the efficiency of the filter attached. Then, a functional fit testing protocol proceeded with testing in four conditions, each lasting 60 s: normal breathing, moving the head from left to right repeatedly, speaking out loud, and finally bending over repeatedly, touching the toes. For each condition, a Fit Factor (FF) was calculated according to the N95 Protocol, and the Global Fit Factor obtained according to the formula:

Global FF = 4/(1/FFa + 1/FFb + 1/FFc + 1/FFd)(2)

For comparison, TIL and FF were measured on a certified FFP2 mask (3M Aura 9322+ FFP2 NR).

### 2.2. Respiratory Safety

#### 2.2.1. End-Tidal CO_2_ Measurements

**Figure 5:**
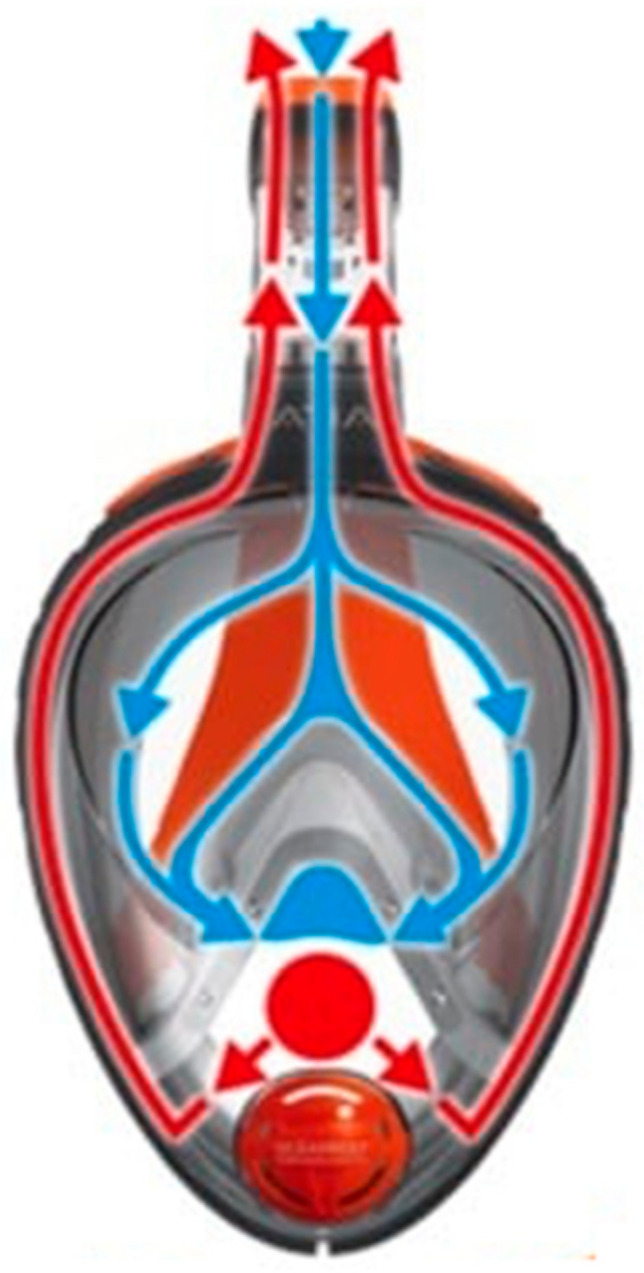
Schematics of air flow in snorkel masks. Blue—inspiration flow, red—expiratory flow.

Respiratory safety was evaluated by measuring simultaneously the end-tidal carbon dioxide (ETCO_2_) levels sampled by a fine collecting tube passed between mask skirt and cheek into the breathing chamber of the mask. This was found not to result in significant leakage. All masks are fitted with a silicone separation with two one-way valves between the nose and the eyes, creating a small oro-nasal internal mask (breathing chamber). Inspired air flows from the inspiratory snorkel channel (situated above the eyes, usually centered on the forehead) toward the breathing chamber (nose and mouth, through the two valves), while during expiration, air flows through dedicated expiration channels laterally toward the snorkel, or through a central ventral valve directly outward (Figure 5). This construction is intended to minimize respiratory ‘dead space’ while at the same time reducing fogging-up of the viewing chamber. The ETCO_2_ level was measured continuously using side-stream measurement, using the medical monitoring system of the hyperbaric chamber at the Military Hospital, Brussels, in atmospheric (1 ATA) conditions (doors open).

Simultaneously, fingertip saturation of oxygen (SaO_2_), heart rate (HR), and transcutaneous CO_2_ (PTcCO_2_) and oxygen (PTcO_2_) levels were measured (HMMS Medical Monitoring System, Haux GmbH, Karlsbad, Germany).

The respiratory safety test protocol consisted of baseline measurements without a mask, followed by two measurements 10 min apart while wearing the mask in rest; then, physical effort was performed, first using only the arms (‘dumbbell’ lifting with both arms of a 6 kg weight), then performing 20 deep knee-bends. Then, 10 min later a resting measurement was taken while still wearing the mask, and finally another baseline measurement without a mask. All measurements were taken as described, except for the baseline ETCO_2_ measurements (without wearing the mask) which were done with a portable main-stream measurement system (Viamed VM2500M, Viamed Ltd., Keighley, West Yorkshire, UK) as without a breathing chamber no side-stream measurement could be taken.

#### 2.2.2. Mask Volume Measurements and Respiratory Dead Space

Respiratory dead space was measured by filling the complete mask and snorkel with water. Dead space was calculated from the volume of the breathing chamber, plus the expiratory channels up till the one-way exhaust valves, if any.

#### 2.2.3. Inspiratory and Expiratory Pressure Measurements

Work of breathing was estimated by measuring inspiratory negative pressure and expiratory positive pressure with a hand-held pressure monitor (DPMIII Universal Biometer, Bio-Tek Instruments, Winooski, VT, USA), both in resting conditions and immediately after physical effort. For this, a small sampling line was introduced in a similar fashion as the ETCO_2_ sampling line.

### 2.3. Practical Aspects

Practical aspects of each mask were evaluated subjectively. Mask wearing comfort is obviously dependent on proper size selection. Visor fogging could be dependent on the efficiency of the air flow inside the mask or the sealing of the inner valves (which prevent humid expired air to flow back onto the visor surface). Spoken communication was tested by judging voice clarity and volume change. Other aspects evaluated were temperature inside the mask, pressure points from the mask skirt, visual field reduction while wearing the mask, and ease of donning and doffing.

## 3. Results

### 3.1. Protection Level

Most masks appeared to provide protection levels within acceptable limits for half-masks. The TIL measured using the normal protocol (non-N95) demonstrated an inward leakage of 15–20% for all masks fitted with an electrostatic respiratory hospital filter; those masks with a makeshift covering of the snorkel opening with FFP2 cloth performed better—close to, or just passing the 5% TIL criteria. Using the N95 protocol, TIL was acceptable for all masks. The functional fit tests (with N95 protocol) were acceptable for all masks with 3D adaptor and filter, and just sufficient for the masks with covered snorkel.

Two exceptions were notable: the Subea mask, despite proper fit, tended to release the upper part of the skirt during Forward Bending, allowing unfiltered air inflow. This is due to the design of the skirt and frame, and has been described elsewhere [16]. The second exception illustrates one risk of the makeshift covering of the snorkel opening with FFP2 cloth: as the Ocean Reef snorkel has lateral grooves on its outer surface, cello-taping this hermetically is very difficult. This creates a significant leak and annihilates the otherwise good scores obtained with the other configurations of this mask. Although a certified FFP2 mouth mask (3M Aura 9322+) yielded better results for the TIL measurements, this mask too was less efficient during normal use and especially during Forward Bending. See Table 2 for complete results.

### 3.2. Respiratory Safety

#### 3.2.1. End-Tidal CO_2_ Measurements

**Figure 6:**
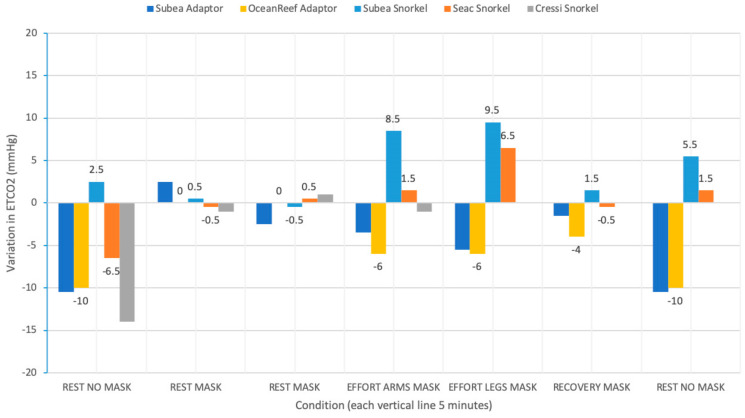
End-tidal CO_2_ (ETCO_2_) variation in various conditions.

After donning the mask, in most masks an increase in ETCO_2_ was measured (Figure 6). The magnitude of this increase cannot be determined with certainty as the two conditions were measured with a different device (main-stream vs. side-stream). The side-stream ETCO_2_ in resting conditions with mask, calculated as the mean of measuring points after 10 and 20 min, was taken as the baseline. Physical effort, first with arms only, then with legs only, resulted either in an increase (Subea A and Seac masks with cloth-covered snorkel), or a decrease of ETCO_2_ (all Subea and Ocean Reef masks with 3D adaptor) probably owing to the generally more favorable breathing characteristics (including a smaller dead space).

The Cressi, Aqualung, and Ocean Reef A (with cloth-covered snorkel) did not allow physical effort as the inspiratory flow was completely blocked by forceful inspiration (blocking of the snorkel tube by the ball/float).

Similar trends were noted with PTcCO_2_ measurement (data not shown).

#### 3.2.2. Oxygen Saturation Measurements

Oxygen saturation remained within physiological limits throughout, and in masks with snorkels even increased a small amount from baseline after donning the mask, probably to a small end-expiratory pressure effect. PTcO_2_ measurements remained stable throughout (data not shown).

#### 3.2.3. Mask Volume Measurements and Respiratory Dead Space

A significant respiratory dead space was present in all snorkel masks. The internal breathing chamber, separated from the eye chamber with one-way valves, appears to be critical—without a proper functioning of those valves, respiratory dead space would increase by more than 800 cm^3^ (Table 3). The exchange of the snorkel with a 3D printed filter adaptor reduced respiratory dead space. The Ocean Reef mask’s 3D accessory is fitted with a one-way inlet valve, modifying the inflow of air to happen not only through the normal inflow channel but also through both expiration channels; expiration takes place exclusively through the frontal (‘water-draining’) valve. This decreases dead space even more and creates a large inspiration channel diameter (at least when used with the 40 mm threaded filter).

#### 3.2.4. Inspiratory and Expiratory Pressure Measurements

As expected, masks with cloth-covered snorkel necessitated, in rest, more negative pressure on inspiration; expiratory values were not much different. With forced respiration, such as after physical effort, masks with cloth-covered snorkel required significant inspiratory effort, in three cases (Cressi, Aqualung and Ocean Reef A) becoming ‘unbreathable’ as the ball/float of the snorkel blocked all air entry. The certified FFP2 mouth mask (3M Aura 9322+) yielded the best results throughout (Table 4).

### 3.3. Practical Aspects

The visor surface of all masks is flat, except for the Aqualung Atlantis 2.0, which is panoramic. This would offer an advantage in water, with better field of view and less distortion, however in air this does not result in a significantly wider field of view (which is almost 180° in all masks). The (transparent) side panels of flat visors cause a certain visual distortion at the edges, and one manufacturer (Ocean Reef) specifically warns about this in their instructions. All of these masks cannot be worn when wearing prescription glasses, not only because of the limited space inside the visor but also because the temples would inevitably break the lateral seal. Ocean Reef provides a prescription glasses insert (as part of their normal snorkel product), and offers several strengths of prescription lenses to fit those. On the internet, glasses inserts for other brands of masks may be found.

Fogging of the visor was not a problem in any of the masks, testifying to the efficient air flow. Likewise, subjective temperature on the face was not uncomfortable, even in direct sunlight and during physical effort.

Spoken communication was slightly impaired, especially for those masks that needed a tight fit and had high inspiratory resistance (Seac, Aqualung and Cressi). Wearing the mask resulted in a muffled sound requiring slow and articulated speech to be understood by bystanders more than one meter away. It is clear that these masks do not allow communication over great distances.

Mask fit is obviously dependent on the shape and size of the wearer’s face. Most masks have a limited number of sizes available (mostly two sizes), only Ocean Reef offers a choice of four sizes. Masks with a smaller rigid outer rim (Aqualung, Cressi) were more difficult to don than those with a larger rim. The strap attachments can be a hindrance to effective donning; Ocean Reef is the only mask where release clips are fitted to the lower end of the straps. All masks are made of lightweight material and weigh between 0.50 and 0.55 kg; the snorkels weigh around 150 g. Those masks that need a tight fit because of slightly inadequate size are obviously less comfortable to wear over longer periods of time, and may cause pressure points after being worn for more than one hour. Clear donning instructions are not always provided in a user manual, however, the Ocean Reef and Subea manuals specify that the chin should be positioned first before securing the straps. The Ocean Reef website has a full section on the configuration of the mask as a personal protective device; for Subea this information is, only in France and Belgium, supplied by the consortium which has created this adaptation (see below).

The Subea masks appeared to be less reliable when performing movements such as forward bending, with at times a wide gap appearing between the upper side of the mask and the forehead. This seems to be due to the design of the skirt, and while it is possible to avoid this by tilting the mask before tightening the head straps, this is by no means straightforward or intuitive.

Facial hair or beard creates a leak and should not be positioned between mask skirt and face.

Both masks for which 3D printed adaptors are available (Subea and Ocean Reef) accommodate 22 mm outlet (ISO 5356-1:2015) hospital filters; Ocean Reef’s adaptor is standard fitting a 40 mm 1/7″ (EN 148-1/STANAG 4155) threaded filter, used in most professional and military gas masks. A low-profile 40 to 22 mm connector piece (also available as a 3D print or from the manufacturer) allows attaching 22 mm filters.

At the time of testing, 3D adaptors for other masks were not available. On the internet, 3D print design files can however already be found, so printing one’s own adaptor would be easy.

The build quality of the Ocean Reef adaptors, as supplied by the manufacturer, was very good. Ocean Reef makes the 3D printing files available free of charge, issuing a warning about the materials to be used. The 3D adaptors for the Subea mask were ‘home printed’ and proved to be much less sturdy: after placing and removing them a few times, cracks appeared and the fixating panel slip broke off. Care must be taken to ensure using the proper materials when ‘home printing’.

The Ocean Reef website contains detailed instructions on cleaning the mask, indicating that it can be submerged in boiling water or placed in a dishwasher on a hot program. All masks appeared to be unscathed after such a (single) treatment. Cleaning the visor and skirts with quaternary ammonium (alcohol-free) disinfectant (spray and wet wipes) did not result in discoloration or opacification of the visor. Cleaning and disinfection of the masks have not been tested for many cycles, so the effect on the acrylic visor window and silicone skirts after repeated cleaning remains to be seen.

## 4. Discussion

Although the possibility of transmission of SARS-CoV2 through other body fluids (such as feces, blood, urine) may be possible (virus particles have been detected in those fluids) [17], aerosol or droplet transmission is considered the most frequent route. Therefore, respiratory and eye protection are considered essential in preventing contamination in healthcare workers who are in direct contact with patients or their respiratory secretions [2,18].

Even if full face mask wearing is not listed among the recommended protection strategies for use during aerosol-generating procedures [19,20], many healthcare workers see the availability of having a single-piece, reusable, eye, nose, and mouth covering protection device as a useful asset. Numerous reports in online newspapers, social media, and on developers’ websites indicate that there is a real interest in their use, and they are already used in many hospitals in Belgium and The Netherlands (and probably elsewhere). The usefulness of such devices may not be limited to in-hospital use such as in emergency wards and operating rooms, but also dental practices, cleaning services, and even out of hospital in emergency medical response teams, or military (para)medical teams. Therefore, we sought to verify some of the claims made in these online reports and ascertain protection level, safety, and advantages and disadvantages of these masks.

Fit testing reports have been made available online, reporting either vastly insufficient [21] or satisfactory fit [6], using similar measuring technology as our method. However, Antwerp Design Factory [21] did not mention using the N95 protocol and furthermore assumed that a certified 99.99% efficient bacterial/viral filter would at least correspond to a FFP3 or N99 filter capacity. From our measurements, it appears that none of the electrostatic filters (Medtronic DAR, Vyaire MicroGard II) passed N95/FFP2 criteria, whereas the mechanical (pleated) PALL BB50T filter performed as good as the P3 filter. The clinical importance of this find cannot be overstressed. Particle count measurement criteria are different from bacterial/viral efficiency measurement criteria [22], both with regards to size and nature of measured particles.

The size of SARS-CoV2 viruses have been measured at 60–140 nanometers [23], and droplet sizes of SARS-CoV2 loaded aerosols have been measured at 0.25–1.0 micrometers (μm) or in the supermicron region (>2.5 μm) [24]. One can expect that any mask/filter combination that blocks those sizes (as measured by the Portacount Pro+ 8038, which measures particles from 0.01 to 1 μm in size) should offer adequate protection from SARS-CoV2 respiratory exposure. However, a filter that effectively blocks out particles according to the N99/FFP3 standard as well as being 99.999% effective in bacterial/viral blocking, would be the logical choice to ensure safety [2]. The P3 filter used has not been formally tested according to the Nelson Labs protocol [22], which leaves the PALL BB50T (the mechanical pleated filter) as the “best choice” in our test. We did not test other pleated mechanical filters, but it is likely that these would, in general, offer better protection than electrostatic filters.

In an attempt to improve the protection factor offered by these masks, recent developments have been reported to add a positive-pressure pump to the snorkel in- and outlet of the Subea mask [25]. However, this modification, while it may provide somewhat better protection, is more expensive, cumbersome, and still does not comply formally with the certification requirements for medical personal protection devices. Proper mask fit without leak appears to be effective without the addition of a positive pressure system. The additional security offered by such systems must be weighed against disadvantages and possibly a false sense of security, as improper fit will also with these systems decrease protection significantly.

Snorkel masks were created for in-water use, whereby a few extra centimeters of water pressure against the mask increase the seal. Additionally, the seal of the silicone skirt on wet skin appears to be better than on dry skin. This makes that proper sealing is not always self-evident. As per some manufacturers’ instructions (Subea, Ocean Reef), when the mask is placed, a fit test can be performed by blocking the snorkel adaptor or filter opening with the palm of one’s hand and inhaling forcefully to verify suction from the mask skirt onto the face. After this, head straps should be adjusted to fit snugly but not too tight. This maneuver was performed prior to formal Fit Testing and resulted in proper fit.

None of these masks have received official clearance for use as PPD; for the Subea masks setup, a French consortium has been created with the manufacturer (Decathlon) and a 3D print design lab (BIC, Clichy, France) to obtain a temporary authorization from the French Department of Medical Devices (ANSM, Saint-Denis Cedex, France: www.ansm.sante.fr) (until 31 May 2020) to market their ‘Pneumask COVID-19′ solution, in view of the current exceptional situation.

The design of all of these masks is roughly similar, however, some differences may be significant.

### 4.1. Expiration Channels

The diameter and location of the main expiration channels may theoretically pose a risk. Most designs incorporate a dual-channel snorkel design, whereby the expiration channel(s) merge with the inspiration channel(s). This creates a risk of CO_2_ recirculation, as exhaled air is (at the top of the snorkel) mixed with fresh inhaled air. Previous tests with the Subea mask [26], have shown that this indeed happens, however, the CO_2_ level in rebreathed did not exceed 2%. The Seac design has the expiration channels exit at the top of the face mask rim, faced backward, and each protected by an exhaust valve. This reduces respiratory dead space as well as protects from CO_2_ rebreathing, however, these valves are not easily accessible nor can they be covered by attaching a FFP2 cloth and may thus, in case of faulty function of those valves, reduce the protection level significantly. The effect of repeated cleaning cycles on these valves remains to be determined. The Ocean Reef design while in origin ‘classic’, is modified by the one-way inlet valve in the 3D adaptor, as described below.

### 4.2. Air Flow Channeling

The design of the 3D printed parts to fit the Subea and Ocean Reef masks is different. The Subea adaptor has both inspiration and expiration channeled through the filter element, thus changing little or nothing to the original breathing gas flow. The Ocean reef adaptor instructions for use include the mounting of a one-way inhalation valve at the filter end, so that expiration through the filter is blocked. Inspiration thus takes place not only through the regular inspiration channel (directed through the visor area and then into the breathing chamber) but also through the regular expiration channels directly leading to the breathing chamber. Expiration can only take place through the frontal (water-draining) valve. While this increases the maximally attainable inspiratory flow, thus permitting a higher level of physical effort, it places more stress on the important single expiratory valve. Ocean Reef also created a 3D printed adaptor for this frontal cover valve, so a similar filter (22 mm connector diameter) can be fitted here. However, this is not part of the standard instructions for setting up this mask as a personal protection device. Fit testing showed that not applying this particular setup did not result in inward mask leak.

### 4.3. Front Valve (Water-Draining Valve)

This valve serves as an outward-only valve, and is intended to facilitate the removal of any water that may have entered the mask during snorkeling. This valve is located immediately in front of the mouth and theoretically, when not sealing completely, can allow unfiltered air to be inhaled. The opening pressure of this valve has been tested on one mask (Subea) by Kothari et al. [16] to be 25 mm of water pressure. While this should be largely sufficient to prevent inadverted opening, valve production quality, degradation after multiple sterilization or cleaning cycles, and any cleanliness issues may present a direct loss of protection. Therefore, a recommendation to cover these valves with extra FFP2 quality cloth, should probably be issued—even if this means extra work to replace that protection cloth during the cleaning process.

When no 3D parts were available, the snorkel opening was covered with FFP2 cloth. For Subea, the snorkel tube could be disassembled to remove the inside ball/float, which ensures protection from water ingress when snorkeling. For the other masks (Seac, Aqualung, Cressi and Ocean Reef), this was not possible as the snorkel tube was glued shut. In three of these masks (Aqualung, Cressi, and Ocean Reef A) the adding of FFP2 cloth to cover the snorkel inlet opening, the smaller diameter, and the increased flow through the snorkel during physical effort caused the ball/float to be aspirated upward and close the inlet opening. This resulted in an impossibility to inhale forcefully, causing acute respiratory distress necessitating immediate removal of the mask. When used in an infectious environment, this obviously would cause immediate exposure to droplets and aerosols, so these masks pose a significant hazard and should not be used. The Seac mask did not cause such an acute inspiratory block, probably due to the heavier weight of its ball/float. However, during exercise the inspiratory flow still required a significant inspiratory effort, causing discomfort and a higher negative pressure.

Even if it would not be hazardous in case of physical effort, such a makeshift FFP2 cloth covering would have to be removed and changed regularly, necessitating considerable manual work and lots of cello-tape. Even then, the design and irregularities of the snorkels would not guarantee a proper hermetically FFP2 cover. We therefore strongly recommend against this because of demonstrated lower protection level, higher work of breathing, and cumbersome mask cleaning and filter changes.

## 5. Conclusions

Snorkel masks, when fitted with a 3D adaptor and a ventilator filter, can provide protection comparable to N95/FFP2 or even N99/FFP3 levels (depending on the type of filter used, electrostatic, or pleated). They should not be used with a makeshift N95/FFP2 covering of snorkel opening, both for reasons of efficiency and safety.

Breathing comfort and safety for both the Subea and Ocean Reef masks with adaptors is adequate, with minimal end-tidal CO_2_ increase at rest and good exercise tolerance.

Mask fit, by selecting the correct size and careful attention to proper donning and positioning, is paramount. Ideally, a ‘fit testing’ should be performed to verify each person/mask combination; however, a rapid ‘suction test’ as described by Ocean Reef, usually allows verification of proper fit. Facial hair (beard) and scalp hair should not be present between skin and mask skirt.

Extra useful features such as an improved respiratory air flow, a 40 mm threaded filter mount, prescription glasses insert, and head strap clips, are as of yet only proposed by Ocean Reef.

These masks, when fitted with a P3 or pleated mechanical hospital ventilatory filter, can serve as a personalized, efficient full-face protection for SARS-CoV2 exposure, and could be considered for use in high-risk circumstances even in the absence of a shortage of ‘classical’ PPD’s (N95 oro-nasal mask, goggles, face shield).

## Figures and Tables

**Table 1:** Snorkel mask configurations tested.

Mask ID	Mask Brand and Type	Snorkel Assembly	Filter Used
Subea A	Subea Easybreath	Original snorkel (*)	FFP2 filter cloth
Subea B	Subea Easybreath	3D printed snorkel adaptor	Medtronic DAR 352U5805
Subea C	Subea Easybreath	3D printed snorkel adaptor	Medtronic DAR 352U5877
Subea D	Subea Easybreath	3D printed snorkel adaptor	PALL BB50T
Subea E	Subea Easybreath	3D printed snorkel adaptor	Vyaire MicroGard IIB
Seac	Seac Unica	Original snorkel (**)	FFP2 filter cloth
Aqualung	Aqualung Atlantis 2.0	Original snorkel (**)	FFP2 filter cloth
Cressi	Cressi Duke	Original snorkel (**)	FFP2 filter cloth
Ocean Reef A	Ocean Reef Aria QR+	Original snorkel	FFP2 filter cloth
Ocean Reef B	Ocean Reef Aria QR+	3D APA snorkel adaptor	Medtronic DAR 352U5805
Ocean Reef C	Ocean Reef Aria QR+	3D APA snorkel adaptor	Medtronic DAR 352U5877
Ocean Reef D	Ocean Reef Aria QR+	3D APA snorkel adaptor	PALL BB50T
Ocean Reef E	Ocean Reef Aria QR+	3D APA snorkel adaptor	SPE P3 R M40040-SP (***)

*: the snorkel’s water entry protection ball/float was removed. **: a 3D printed adaptor for this mask could not be sourced. ***: this adaptor provides for a 40 mm threaded connector, allowing the fit of professional filters. Note: the Vyaire MicroGard II filter does not have a 22 mm connector and cannot be fitted to the Ocean Reef adaptor.

**Table 2:** Protection levels as measured by Total Inward Leakage (TIL) and Fit Factors.

Mask ID	TIL	TIL (N95)	FF NB	FF Lat	FF Talk	FF Bend	FF Overall
Subea A	6.84%	1.56%	105	97	114	25	58
Subea B	16.65%	0.06%	200+	200+	200+	200+	200+
Subea C	17.92%	0.05%	200+	200+	200+	200+	200+
Subea D	0.09%	0.05%	200+	200+	200+	200+	200++
Subea E	19.15%	0.04%	200+	200+	200+	16	52
Seac	4.11%	0.09%	200+	200+	200+	200+	200+
Aqualung	3.11%	0.33%	168	142	121	77	117
Cressi	6.51%	1.09%	192	183	171	112	157
Ocean Reef A	18.74%	0.97%	84	63	48	47	57
Ocean Reef B	15.17%	0.05%	200+	200+	200+	200+	200+
Ocean Reef C	18.43%	0.03%	200+	200+	200+	200+	200+
Ocean Reef D	0.02%	0.07%	200+	200+	200+	200+	200+
Ocean Reef E	0.06%	0.03%	200+	200+	200+	200+	200+
3M Aura 9322+ FFP2	0.10%		97	112	126	27	62

TIL = Total Inward Leak; FF = Fit Factor; NB = Normal Breathing; Lat = moving head from side to side; Talk = talking out loud; Bend = forward bending touching toes.

**Table 3:** Mask volumes and respiratory dead space.

Mask	Face/Eye Volume (cm^3^)	Nose/Mouth Volume (cm^3^)	Snorkel/Adaptor Volume (cm^3^)	Total Volume (cm^3^)	Respiratory Dead Space (cm^3^)	Inspiratory Diameter (cm^2^)
Subea Easybreath M/L (snorkel)	800	250	180	1230	469	2.7
Subea Easybreath M/L (adaptor)	800	250	30	1080	380	1.9
Seac Unica M/L (snorkel)	850	250	100	1200	350	5.76
Aqualung Atlantis 2.0 L/XL (snorkel)	370	200	150	720	399	2.16
Cressi Duke S/M (snorkel)	850	400	220	1470	610	5.04
Ocean Reef Aria L (snorkel)	825	250	180	1255	250	2.8
Ocean Reef Aria L (adaptor)	825	250	70	1145	250	1.9 (22 mm) or 4.2 (40 mm)

**Table 4:** Inspiratory (‘Insp’) and expiratory (‘Exp’) pressures (in cmH_2_O).

Mask Brand and Type	Normal Insp	Normal Exp	Forced Insp	Forced Exp
Subea A	−4	+1	−13	+4
Subea B	−4	+2	−11	+4
Subea C	−2	+2	−9	+3
Subea D	−2	+1	−11	+4
Subea E	−1	+1	−6	+3
Seac	−6	+2	−21	+6
Aqualung	−6	+2	−26 *	+7
Cressi	−5	+2	−33 *	+6
Ocean Reef A	−7	+2	−35 *	+3
Ocean Reef B	−5	+2	−13	+4
Ocean Reef C	−2	+1	−11	+3
Ocean Reef D	−2	+1	−11	+3
Ocean Reef E	−2	+1	−8	+4
3M Aura 9322+ FFP2	−1	+1	−3	+2

*: inspiration impossible due to blocked snorkel.
